# Kink pair production and dislocation motion

**DOI:** 10.1038/srep39708

**Published:** 2016-12-22

**Authors:** S. P. Fitzgerald

**Affiliations:** 1 Department of Applied Mathematics, University of Leeds, Leeds LS2 9JT, UK

## Abstract

The motion of extended defects called dislocations controls the mechanical properties of crystalline materials such as strength and ductility. Under moderate applied loads, this motion proceeds via the thermal nucleation of kink pairs. The nucleation rate is known to be a highly nonlinear function of the applied load, and its calculation has long been a theoretical challenge. In this article, a stochastic path integral approach is used to derive a simple, general, and exact formula for the rate. The predictions are in excellent agreement with experimental and computational investigations, and unambiguously explain the origin of the observed extreme nonlinearity. The results can also be applied to other systems modelled by an elastic string interacting with a periodic potential, such as Josephson junctions in superconductors.

Dislocations are ubiquitous line-like crystal defects whose motion gives rise to plastic deformation^1^,^2^. Their dynamics controls the mechanical properties of crystalline materials such as strength and ductility, and understanding this is crucial to predict the behaviour of structural and functional materials for many applications. An elastic string moving through a periodic potential provides an idealized model for a multitude of physical systems, including coupled torsion pendula, crowdion defects, and vortices in superconductors^3^,^4^,^5^,^6^, as well as dislocations. In the context of dislocation theory, the periodic lattice potential is known as the *Peierls potential*, and the critical applied bias above which no minima exist is called the *Peierls stress*. Below this value, dislocation motion, and hence plastic deformation, is possible via the thermal escape of the dislocation from a metastable well, as depicted schematically in Fig. 1. The rate for this process depends on the applied force in a highly nonlinear way, and has been measured in iron^7^ to be proportional to up to the fortieth power of the applied stress. Such extreme nonlinearity is intriguing, and of great practical importance. Many efforts have been made to calculate the rate theoretically and numerically for the general system^4^,^8^,^9^, and for dislocation dynamics in particular^10^. Dorn and Rajnak^11^ assumed a double-sine form for the Peierls potential and an Arrhenius form for the rate, successfully capturing the dislocation response in a range of materials. Edagawa *et al*.^12^ extended the model to two dimensions. Quantum mechanical effects can also be important^13^,^14^. The paper is organized as follows. In section 2, we use path integral techniques to show that the kink pair nucleation rate, as an Arrhenius-like form in the saddle point energy, can be derived directly from the driven, overdamped sine-Gordon equation for an elastic string. The saddle point energy is derived as an integral of the square root of the potential, in analogy with results from soliton theory. The exact expression holds for any profile of the Peierls potential, and is not restricted to a sine or double-sine form. Section 3 assumes a sinusoidal Peierls potential and derives an approximate fully analytical expression (Eq. 15) for the dislocation velocity as a function of applied stress, which is suitable for implementation in dislocation dynamics simulations. Finally in Section 4 we compare our results to existing expressions based on elasticity theory, experimental data, and molecular dynamics (MD) simulations, and present conclusions in Section 5. The analysis applies equally to any overdamped system that can be modelled by Eq. (1) below.

## Derivation of exact rate

The dislocation is modelled as an elastic string moving in a biased periodic potential (line tension approximation). Elasticity theory is not used, and an effective exponential interaction between the kinks emerges (Eq. 14). Though the edge (screw) nature of kinks on screw (edge) dislocations is neglected, atomistic studies have shown that, as far as kink energies are concerned, the line tension approximation is adequate (see the discussion following Eq. (1) in refs 15 and 16). The model’s deterministic, continuum equation of motion takes the general form


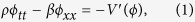


a wave equation for the string plus a forcing term from the negative gradient of 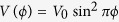, for example. *ρ* is the string’s density, *β* its tension, and *V*_0_ is the magnitude of the lattice potential, whose period is taken to be 1. Since the applied stresses we consider are too low to qualify as shock loading, the motion of the dislocation line is overdamped, and inertia can be safely neglected (see ref. 17 for a treatment of inertial effects). Removing the inertia term from Eq. (1), adding dissipation Γ*ϕ*_*t*_, noise *ξ*(*x*, *t*), and bias *F* so that 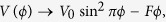 leads to the Langevin equation


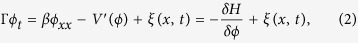


where


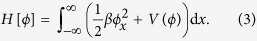


The simplest choice for *ξ* is *Gaussian white noise*, with correlation function 〈*ξ*(*x*, *t*)*ξ*(*x*′, *t*′)〉 = 2*Dδ*(*x* − *x*′)*δ*(*t* − *t*′), and probability distribution


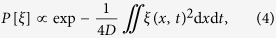


and the noise strength *D* = Γ*k*_B_*T* by the fluctuation-dissipation theorem. Eq. (4) can be transformed into a distribution for string configurations *ϕ* by substituting *ξ* according to Eq. (2):


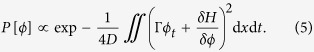


(This neglects the functional Jacobian of the transformation, which is permissible if Eq. (2) is interpreted as an Îto stochastic differential equation. This depends on the underlying discretization of the coordinates, and may need to be modified if higher order corrections to the rate are computed. Here only the leading non-perturbative contribution is considered, and the Jacobian is irrelevant^18^). The integral over *x* and *t* in Eq. (5) above is a generalized Onsager-Machlup action *S*[*ϕ*] = ∫*L*(*ϕ*_*t*_, *ϕ*_*xx*_, *ϕ*), and plays a role analogous to the classical action in quantum field theory. The transition rate from the metastable well to the stable one can be written as


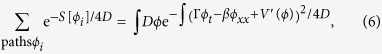


where the functional integral is taken over all field configurations *ϕ*_*i*_ that satisfy the initial and final conditions. Proceeding in analogy with quantum field theory, the integral will be dominated by configurations that extremize *S*[*ϕ*], i.e. those which satisfy its Euler-Lagrange equation


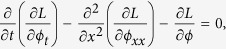


which can be written as


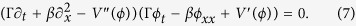


This is clearly solved by *ϕ*^−^ satisfying


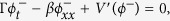


which is simply the noiseless version of the equation of motion, (2). This has action *S*^−^ = 0, and corresponds to the motion from the saddle point to the potential minimum, for which fluctuations are not required^19^. Eq. (7) is also solved by *ϕ*^+^ satisfying


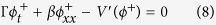


− compare the inverted potential which emerges in instanton calculations in Euclidean quantum field theory^20^. It corresponds to motion *against* the potential gradient, i.e. from the minimum to the saddle point, and its action *S*^+^ quantifies the fluctuations required to surmount the barrier, and hence the rate.

**Figure 1:**
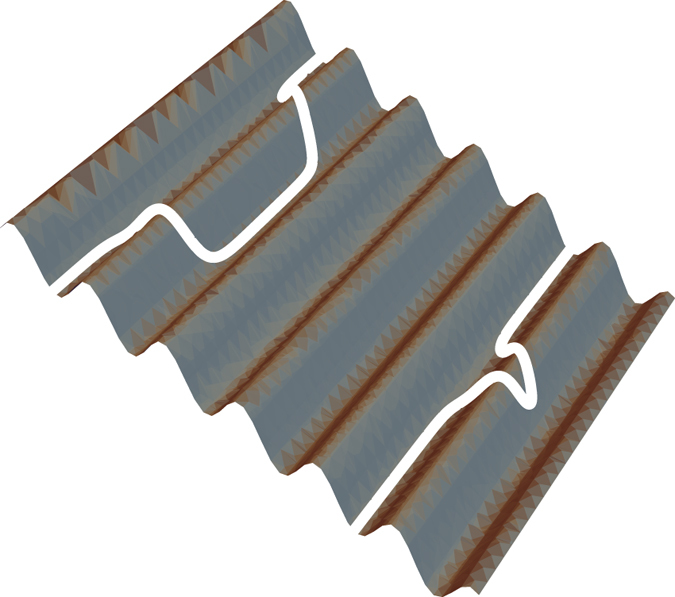
(Schematic, not to scale) Two saddle point configurations for different applied forces. Left: a well-separated kink pair, the critical configuration at small applied force. Right: a small protuberance whose maximum extent does not reach the neighbouring potential minimum, the critical configuration when the applied force nears the Peierls force.

Using the equation of motion for *ϕ*^+^ (8), the action can be determined^19^:


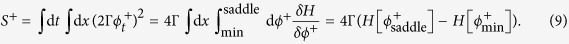



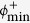
 and 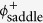 are the initial (minimum) and saddle (maximum) energy string configurations respectively, and the potential can be shifted so that 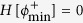. The task remains to determine 
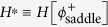
, and it turns out that this can be done exactly, without explicit knowledge of the field configuration 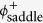. The problem is to maximize *H*[*ϕ*] over fields *ϕ*(*x*) that lie in a potential minimum *ϕ* = *ϕ*_0_, say, as *x* → ±∞, and contain a protuberance out of the minimum and some distance over the potential barrier towards the neighbouring well. For *F* small in comparison to *V*_0_ (i.e. *F* ≪ *πV*_0_), this will be a well-separated kink pair, Fig. 1(left), more of which below, whilst for *F* approaching *πV*_0_, it will be a small bump whose maximum extent *ϕ* = *ϕ*_1_ need not reach the neighbouring minimum at all, Fig. 1(right). In any case, we can assume that the protuberance is centred on *x* = 0, is even in *x*, and satisfies the Euler-Lagrange equation for *H*[*ϕ*]:


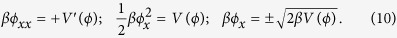


This allows us to write


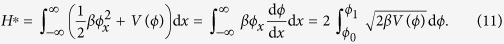


**Figure 2:**
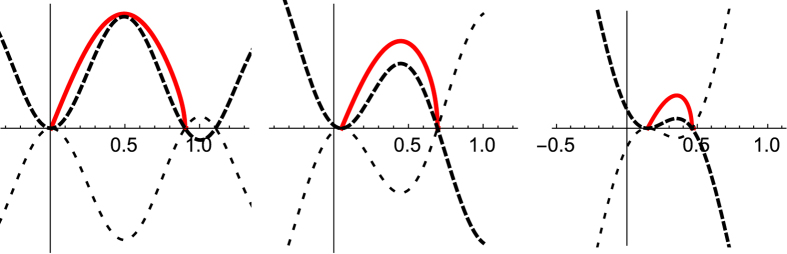
Potentials for small, medium, and large applied forces (left to right). Dashed line: potential *V*(*ϕ*). Dotted line: inverted potential −*V*. Solid line: square root of the potential. The area under this curve is the saddle point energy (see text).

**Figure 3:**
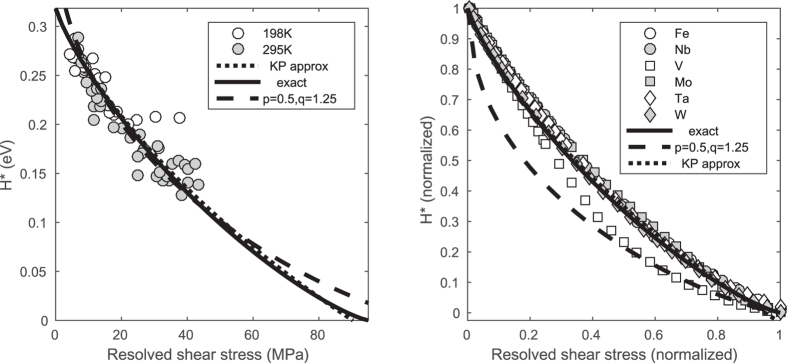
Saddle point energy as a function of the applied force. Left (points): data for edge dislocations taken from Fig. 7 of ref. 27. Right (points): numerically determined energies taken from ref. 15. Solid curves: exact result Eq. (12); dotted curves: kink pair approximation, Eq. (15) (fitted with *E*_K*P*_ = 0.32 eV, *πV*_0_/|*b*| = 95 MPa); dashed curves: *H*_0_(1 − *F*^0.5^)^1.25^.

Now, since *ϕ*_*x*_ = 0 at *ϕ*(0) = *ϕ*_1_, Eq. (10) tell us that *V*(*ϕ*_1_) = 0 also, so we simply need to integrate the square root of the potential between the zero at its initial local minimum, *ϕ*_0_, and its next zero. This is again precisely analogous to the quantum-mechanical instanton calculation, where one imagines a particle rolling from a local maximum in the inverted potential, through a minimum, to a position of equal height further along, before returning to its starting point. This is illustrated in Fig. 2 for small, medium and large values of the applied force. The dashed line is the potential *V*(*ϕ*), the dotted line is the inverted potential −*V*, and the solid line is the (scaled) square root of the potential. The saddle point energy which determines the rate is the area under this curve. This is essentially equivalent to Eq. (23) of ref. 11, but requires no assumptions on the form of the potential. Figure 3 shows H^*^ as a function of the applied force (solid curve), along with an approximation (dashed curve) to be discussed below. Since *S*/4*D* = *H*/*k*_B_*T*, the rate can be written as


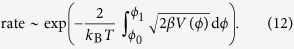


The Arrhenius form derives from the Gaussian white noise, and the explicit damping parameter Γ has cancelled. The 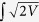 is analogous to similar expressions arising in the WKB approximation, and indeed in soliton theory^21^. The analysis above does not require integrability, and shows that this form for the rate survives in a driven, dissipative system. This expression is exact as far as the potential is concerned, and holds for any value of *F* between zero and the Peierls force *V*_0_*π*. It does not require the explicit form of the saddle point configuration, and hence avoids the need for eigenfunction expansions, or other sophisticated approximation techniques. It can immediately be applied to more complex potentials than the sinusoidal one considered here, e.g. the double-sine form employed in ref. 5 or the computed potentials in refs 15 and 22, and reduces to the results in refs 9, 11 and 23 for appropriate choices of *V*. It is valid at temperatures *k*_B_*T* < H^*^, corresponding to the tree-level Feynman diagrams in quantum field theory. This means it will break down as *F* → *V*_0_*π* and H^*^ → 0. Loop corrections, corresponding to larger thermal fluctuations, may be possible if not analytically tractable. For many metals the above rate will be accurate for applied stresses not too close to the Peierls stress, since H^*^ at zero *F* is of order 1 eV^15^.

## Fully analytical approximation

Eq. (12) is exact and can be easily evaluated numerically for any *F*. However, a simple fully analytical expression would be more helpful for use in dislocation dynamics simulations, as well as providing greater insight into the origin of the nonlinearity in the observed rate. This can be obtained by inserting into *H*[*ϕ*] the approximate solution for a sine-Gordon kink-antikink pair separated by *R*:


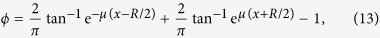



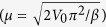
 which leads to


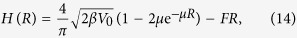


**Figure 4:**
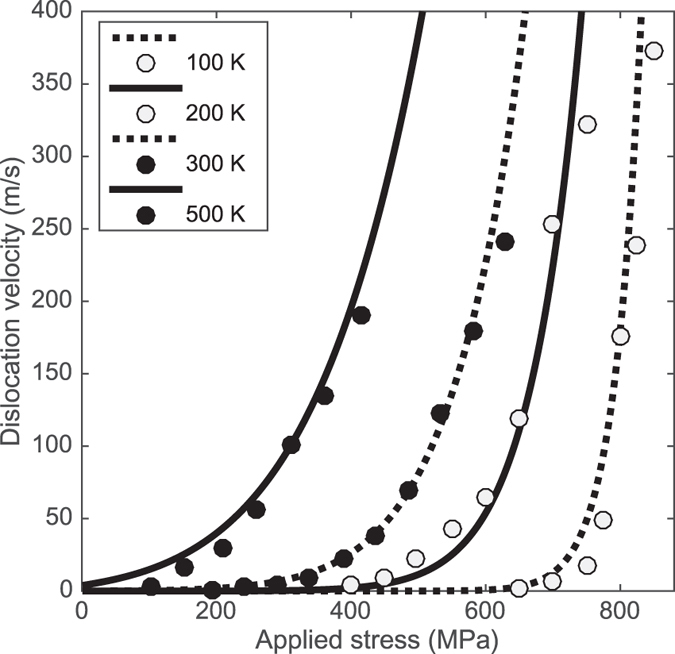
Points: screw dislocation velocities from MD^25^. Curves: *A*exp(−H^*^/*k*_B_*T*) fitted to the 300 K data (see text).

The three terms correspond to the kink ‘rest energy’, the kink-antikink interaction energy, and the energy gain from the applied bias. Maximizing this expression over *R*, and writing 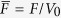 gives


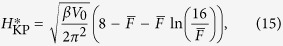


in agreement with the expression presented in ref. 23. H^*^ has been determined numerically using density functional methods (DFT) for the bcc transition metals^15^ (see in particular Fig. 4 in), and the calculation above is the analytical equivalent of the procedure adopted there, where a kink pair ansatz was iterated numerically until converged to the extremal configuration. H^*^ has also been computed using nudged elastic band simulations for W^22^.

## Discussion


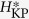
 is shown as dotted curves in Fig. 3 with 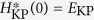, the energy of a fully-separated kink pair, set to 0.32 eV. The shape of the curve is in excellent agreement with the numerical results cited above, and the subtle differences may be ascribed to the non-sinusoidal Peierls potentials in real metals. This approximation should hold in the limit *F* → 0, yet it captures the exact behaviour remarkably well. Indeed, it only fails when *F* is within a few per cent of the Peierls force, where the Arrhenius “exp − *E*/*k*_B_*T* ” form is no longer accurate anyway, obviating the need for a large *F* approximation. This is fortuitous, and is due to the sources of inaccuracy compensating each other to some extent. It is now clear how the extreme nonlinearity in the stress dependence of the rate arises: when exponentiated, the second term will become a factor ~*F*^*F*^ raised to some power. Thus a power series expansion of the rate for small applied forces is not appropriate, and explains the enormous exponents mentioned in the introduction. Especially at low temperatures, the rate resembles a threshold (see Fig. 4 below), and is never linear in the force, however small the force is taken to be. This mathematical effect is not captured by existing nonlinear forms for H^*^ such as the elasticity-derived expression *H*_0_(1 − *F*^*p*^)^*q* ^24,25.

Dislocation velocities are difficult to measure experimentally, and either require *in situ* straining in the transmission electron microscope^26^, where the value of the applied stress is hard to determine, or rely on indirect measurements such as slip band growth^27^. The curves in Fig. 3 (left) are fitted to two datasets for edge dislocations in *α*-Fe taken from ref. 27 by assuming the dislocation velocity takes the form *A*exp(−H^*^/*k*_B_*T*). The fit is certainly consistent, though caution is required, since the data are taken directly from the log-log plot presented in ref. 27. The values of the Peierls stress and the kink pair energy are somewhat lower than those predicted by DFT^15^, though these DFT calculations were for screw dislocations, and this is not unusual in DFT calculations of these quantities in general (see the discussion in ref. 15). The elasticity expression, with *p*, *q* = 0.5, 1.25, is shown as dashed lines in Fig. 3. Both forms are consistent with the experimental data, though they require different values for the Peierls potential and kink pair energy. Figure 3 (right) compares the analytical predictions with the enthalpies determined numerically in ref. 15. With the notable exception of vanadium, the curves are in excellent agreement. In this case, the elasticity result cannot be adjusted since the values of the Peierls stress and kink pair energy are known *a priori*. Given the experimental errors involved, the order-of-magnitude agreement seen here is as good as can be expected.

Extensive molecular dynamics simulations of screw dislocations in *α*-Fe have been performed by Gilbert *et al*.^25^. Fitting *A*exp(−H^*^/*k*_B_*T*) to the 300 K data, and changing only the temperature, results in the curves of Fig. 4. The shapes of the curves, and the temperature-dependence, are in excellent agreement with Fig. 4 of ref. 25. This fit is overly constrained, since as temperature increases, the Peierls potential *V*_0_ and string tension *β* will change as the elastic moduli of the crystal soften. Also, the prefactor *A* will be weakly temperature-dependent (though its calculation from quadratic fluctuations implicit in Eq. (6) requires the evaluation of the determinant of a partial differential operator, and is deferred to a future publication). Varying the parameters independently allows a superior fit, though some caution is required. Subtleties arise in the definition of the Peierls stress, e.g. from non-Schmid effects in the applied stress, and changes in the shape of the periodic potential under stress (see ref. 28 and the discussion and references in ref. 25). The functional form of Eq. (15) is clearly appropriate for describing the experimental and numerical results, and for practical purposes such as implementation in discrete dislocation dynamics simulations, a dislocation velocity rule could be obtained by fitting *A*exp(−H^*^/*k*_B_*T*) to whatever data is available appropriate to the conditions of the simulation.

## Conclusions

A path integral approach was used to derive a simple, general, and exact expression for the kink-pair nucleation rate in sine-Gordon and related systems. Derived directly from the Langevin equation for the elastic string, it can be immediately extended to non-sinusoidal potentials. The approach exploits the well-known analogy between the quantum mechanics of an *n*-dimensional object and the statistical mechanics of an (*n* + 1)-dimensional one. In this *n* = 0 case, the stochastic behaviour of the elastic string enjoys much of the analytical tractability of the quantum mechanical particle. The result can be fitted accurately to numerical and experimental investigations of dislocation mobility, providing an analytical velocity–stress(*σ*)–temperature(*T*) rule suitable for use in mesoscale simulations, which takes the general form


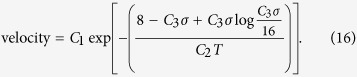


The analysis also applies to the many other stochastic systems modelled by an elastic string in a periodic potential.

## Additional Information

**How to cite this article**: Fitzgerald, S. P. Kink pair production and dislocation motion. *Sci. Rep.* **6**, 39708; doi: 10.1038/srep39708 (2016).

**Publisher's note:** Springer Nature remains neutral with regard to jurisdictional claims in published maps and institutional affiliations.
